# Capgras syndrome associated with limbic encephalitis in a patient with diffuse large B-cell lymphoma

**DOI:** 10.1590/S1980-57642016DN10100012

**Published:** 2016

**Authors:** Herval Ribeiro Soares, Wagner Cid Palmeira Cavalcante, Sebastião Nunes Martins, Jerusa Smid, Ricardo Nitrini

**Affiliations:** 1 Department of Neurology, University of São Paulo, São Paulo SP, Brazil; 2 Department of Pathology, University of São Paulo, São Paulo SP, Brazil

**Keywords:** Capgras syndrome, limbic encephalitis, lymphoma, síndrome de Capgras, encefalite límbica, linfoma

## Abstract

We report the case of a patient with insidious onset and slowly progressive cognitive impairment, behavioral symptoms, temporal lobe seizures and delusional thoughts typical of delusional misidentification syndromes. Clinical presentation along with extensive diagnostic work-up revealed limbic encephalitis secondary to diffuse large B-cell lymphoma. The patient underwent immunotherapy with high-dose corticosteroid but no significant improvement was observed. No specific treatment for lymphoma was performed because the patient died of septic shock following a nosocomial respiratory infection. Delusional misidentification syndromes are an unusual and unique form of cognitive impairment in which a patient consistently misidentifies persons, places, objects, or events. Capgras syndrome is the most common subtype of this disorder, being defined by the recurrent and transient belief that someone close has been substituted by an imposter. These entities are generally associated with neurodegenerative diseases and psychiatric disturbances. Rare reports of associations between misidentification syndromes and autoimmune diseases such as multiple sclerosis have been published, but no papers address a correlation with limbic encephalitis or lymphoma.

## RESUMO

Nós relatamos o caso de um paciente com início insidioso e lentamente progressivo de declínio cognitivo, sintomas comportamentais, crises convulsivas do lobo temporal e pensamentos delirantes típicos dos delírios monotemáticos de identificação. A apresentação clínica associada aos exames complementares levaram ao diagnóstico de encefalite límbica relacionada a linfoma difuso de grandes células B. O paciente foi submetido a imunoterapia com altas doses de corticoide sem melhora clínica. Não foi realizado tratamento específico para o linfoma em função do paciente ter evoluído para óbito por choque séptico secundário a infecção respiratória nosocomial. Os delírios monotemáticos de identificação representam uma forma única e incomum de comprometimento cognitivo nas quais o paciente consistentemente identifica de forma errônea pessoas, lugares, objetos ou eventos. A síndrome de Capgras é o subtipo mais comum dessas desordens, sendo definida como a crença recorrente e transitória que um impostor substituiu uma pessoa familiar. Essas entidades são geralmente relacionadas com doenças neurodegenerativas e distúrbios psiquiátricos. Raros relatos da associação entre os delírios monotemáticos de identificação e doenças autoimunes como esclerose múltipla foram publicados, porém não há artigos abordando a correlação com encefalite límbica ou linfoma.

## INTRODUCTION

Delusional misidentification syndromes (DMS) are an uncommon group of disorders characterized by delusional beliefs that the people, objects, or places around the patient change or are made to change with one another.^1^ The most frequent DMS is Capgras Syndrome (CS), which is defined by the recurrent and transient belief that a person, usually someone closely related, has been substituted by an imposter with very similar features as the original person.^2^ Lesions found in DMS are usually bifrontal or right hemispheric, resulting in disconnection between the frontal lobes and the right temporolimbic regions, which are necessary for reconciling information about self-identification of the person and their associated emotions.^3^ These syndromes are generally associated with neurodegenerative illnesses and psychiatric disorders,^4^ and rarely with other entities such as autoimmune diseases.^5^

Limbic encephalitis (LE) is an uncommon inflammatory and autoimmune neuropsychiatric condition affecting the medial temporal lobe of the brain characterized by subacute cognitive symptoms, short-term memory loss, seizures, and affective changes.^6^ This condition was initially described as a paraneoplastic syndrome of the central nervous system.^6^ Tumors generally associated with LE are small cell lung cancer, breast cancer, testicular tumors, teratoma, Hodgkin's lymphoma and thymoma.^7^ Other types of cancer such as non-Hodgkin lymphomas are rarely reported to be associated with this paraneoplastic syndrome.^8^

We describe the case of a patient who presented with the clinical picture of a DMS which, following a thorough investigation, was found to have LE associated with Diffuse Large B Cell Lymphoma.^9^ To our knowledge, there are no reports in the medical literature associating DMS with LE or non-Hodgkin lymphoma.

## CASE REPORT

A 64-year-old right handed male with a university degree in arts presented to the neurology service with the chief medical complaint of behavioral changes slowly progressing over the course of the past seven years. Initially the patient experienced an insidious onset of decline in work productivity and gradually stopped working within the space of a year. Meanwhile, his family also noticed a short-term memory deficit primarily evidenced by difficulty remembering messages he was supposed to give to his wife, as well as not being able to name some specific objects. In the ensuing years the symptoms kept worsening while new behavioral and motor changes such as apathy, irritability, aggressiveness, delusional thoughts, psychomotor and gait slowing were contributing to progressive cognitive impairment. There were also reports of paroxysmal events characterized by self-limited episodes of experiencing unpleasant smells that lasted for seconds to a few minutes. 

The patient had consulted several physicians during the course of the disease. He was diagnosed with bipolar disorder by a psychiatrist with the report of accelerated thinking at the time, and was treated with olanzapine and valproate without any significant improvement. A neurosurgeon diagnosed normal pressure hydrocephalus and performed a ventriculoperitoneal shunt with no apparent symptomatic relief.

At a later stage, he presented to the Neurology Department of the University of São Paulo with the main complaint of worsening delusional thoughts. The patient stated that his wife had been substituted by a perfect copy of herself, that the house he was living in only resembled his original property, and the city's most famous avenue had been duplicated in two identical copies. 

Past medical history was relevant for Pulmonary Abscess, Hypertension, Depression, Diabetes and former tobacco and alcohol abuse. He had an unremarkable family history. Prescribed medications were Carbamazepine, Olanzapine, Sertraline and Clonazepam.

Neurological examination was remarkable for a cognitive impairment characterized by dysexecutive syndrome (inappropriate digit span, low fluency verbal test, impairment of abstract thinking and problem solving, inadequate clock drawing test), psychomotor slowing and inappropriate behavior. The Mini-Mental State Examination test score was 26 out of 30 (dropped 1 point in spatial orientation and 3 in recall). Glabellar and palmomental primitive reflexes were markedly present as well as inhibitory paratonia, demonstrating frontal release signs. 

Based on initial impressions, the clinical presentation was attributed to LE (short-term memory impairment, temporal lobe seizures and behavioral abnormalities) of protracted course associated with a delusional misidentification syndrome (Capgras Syndrome concomitant to Reduplicative Paramnesia).

The patient was then admitted to the neurology ward for extensive investigation. General laboratory tests were unremarkable, serology for common infectious diseases were negative (HIV, Syphilis, Lyme, Cytomegalovirus, Herpes Simplex), Antinuclear Antibodies (ANA) were positive in high titers (1:2560) but the remaining rheumatologic panel was unrevealing (AntiSm, Anti-Ro, Anti-La, Anti-dsDNA). Brain Magnetic Resonance Imaging (MRI) revealed high signal intensity on T2/ FLAIR in the left temporal pole and left mesial temporal region. Cerebral spinal fluid (CSF) was relevant for moderate lymphocytic pleocytosis (85 cells/mm^3^), elevated protein levels (116mg/dl), normal glucose levels (56 mg/dl), negative flow cytometry for malignant cells, and negative infectious markers (cultures for bacteria and fungi, Syphilis, Tuberculosis, Herpes Simplex, Varicella Zoster, Cytomegalovirus, Epstein Barr Virus). Positron Emission Tomography (PET) with fluorodeoxyglucose (FDG) disclosed hypermetabolism in the left mesial temporal region and peri-insular cortex, as well as severe and diffuse hypometabolism in frontal, parietal and lateral temporal cortices. Electroencephalography showed diffuse slow waves (theta and delta) without epileptiform activity. 

While the aforementioned initial work-up was being performed, the patient was started empirically on acyclovir, ampicillin and fluconazole for 14 days. Following the negative results for infectious agents associated with the clinical picture, temporal lobe abnormalities on advanced neuroimaging studies and poor response to antimicrobials, the hypothetical diagnosis of Autoimmune LE was strengthened. The patient received methylprednisolone 1 gram daily for five days without significant improvement and investigation for a paraneoplastic syndrome was continued. Tests for autoantibodies associated with neurological paraneoplastic syndromes in serum and CSF were negative (Anti-Hu, Anti-Ri, Anti-Yo, Anti-Ma2, Anti-NMDAR, Anti-VGKC, Anti-GAD, Anti-TPO, Anti-TG). Computed Tomography (CT) of the neck, thorax, abdomen and pelvis disclosed no significant findings. Ultrasound of the testicles was also normal. However, whole-body FDG-PET revealed an anomalous increase in glycolytic metabolism in cervical external inguinal and iliac portacaval lymph nodes. A cervical lymph node biopsy was performed but anatomopathological study showed nonspecific neutrophilic inflammatory reaction and absence of signs suggestive of malignancy. The patient was then discharged for clinical follow-up scheduled over the next few months.

Two months following previous hospital admission, the patient exhibited progressive worsening of cognitive and behavioral symptoms and therefore another work-up for occult malignancy was undertaken. The repeat whole-body CT scan disclosed enlarged mediastinal, hilar, cardiophrenic and axillary lymph nodes. The hypothesis of lymphoproliferative disease was proposed and the patient was admitted to the hematology ward for fine-needle aspiration of the axillary lymph node. Papanicolaou and Giemsa-stained cytology slides, and the cell block preparation from the sample material, showed morphologic features of a large cell lymphoma: intermediate-to-large sized cells, nuclei of different sizes and shapes, clumping artifact of naked nuclei, frequent mitosis and apoptotic bodies. Immunophenotyping showed a B-cell phenotype with a high Ki-67 labelling index (Figure 3). The tumor cells were negative for CD 3, CD 30 and pan-cytokeratin AE1/AE3. In light of these immunohistochemical results and morphologic features, the diagnosis of diffuse large B-cell lymphoma was reached. After the procedure, the patient acquired a nosocomial respiratory infection followed by septic shock and evolved to death.

**Figure 1.**
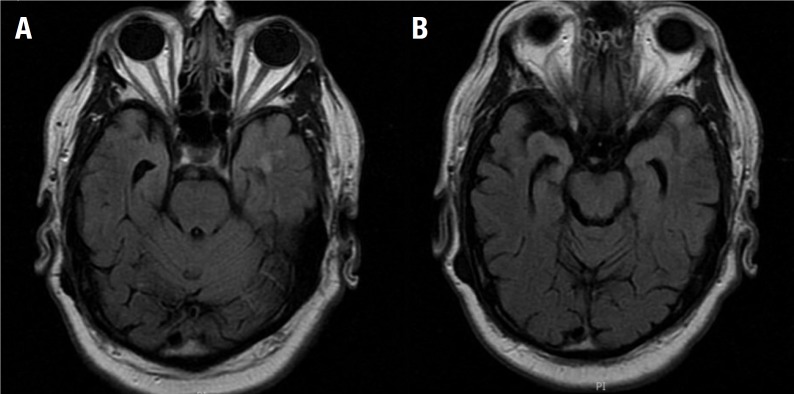
Brain Magnetic Resonance Imaging FLAIR sequence revealing elevated signal intensity in left mesial temporal region [A] and in left temporal pole [B].

**Figure 2.**
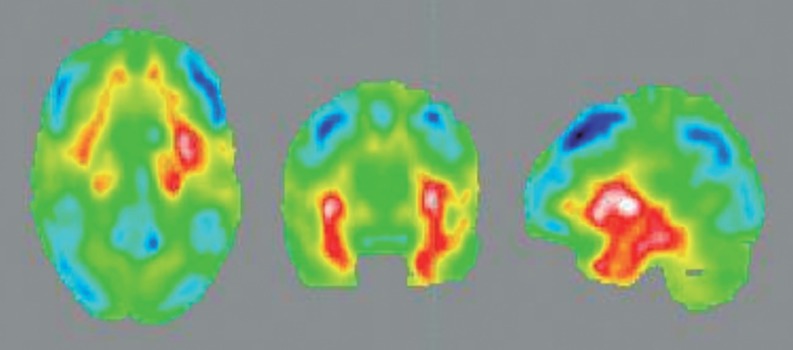
Positron Emission Tomography with fluorodeoxyglucose (PET-FDG) showing increased metabolism in left mesial temporal region and peri-insular cortex (red). Severe and diffuse hypometabolism in frontal, parietal and lateral temporal cortex is also demonstrated (blue).

**Figure 3.**
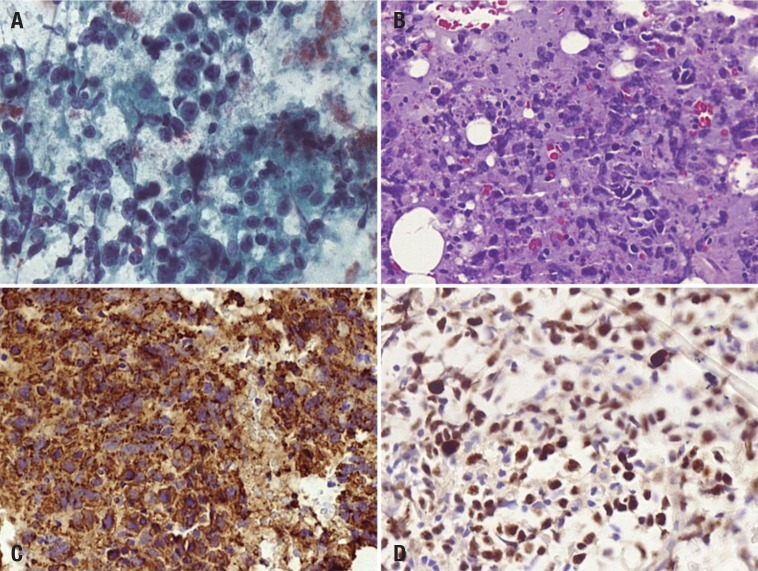
Fine-needle aspiration cytology of the axillary lymph node was diagnostic for Diffuse Large B Cell Lymphoma. [A] FNAC of the nodule showing intermediate-to-large sized cells with prominent central nucleoli and numerous apoptotic bodies (Papanicolaou stain, x400). [B] Cell block preparation from the aspirated material showing similar cellular features (Hematoxylin & Eosin stain, x400). The tumor cells were positive for CD 20 [C] and had a high Ki-67 [D] labelling index (Immunoperoxidase stain, x400).

## DISCUSSION

Delusional Misidentification Syndromes (DMS) are among the most fascinating and puzzling forms of cognitive problems that can result from psychiatric or neurological disease.^10^ They are a psychopathologic phenomena in which a patient consistently misidentifies persons, places, objects, or events.^11^ There are several clinical subtypes of DMS such as Capgras syndrome, reduplicative paramnesia, Fregoli delusion, Cotard syndrome, intermetamorphosis, reverse-intermetamorphosis, and misidentification of reflection.^12^ Despite distinctive differences, these phenomena seem to share the core feature of a profound change in perception of reality and may also share a common neurobiological substrate.^4^ Capgras syndrome, the most frequent variant, is characterized by the delusion that identical-likeness impostors have substituted familiar people.^13^ The subjective belief that a place has been duplicated, existing in at least two locations simultaneously, is termed reduplicative paramnesia.^14^ In the Fregoli delusion, the patient believes that various strangers are actually a familiar person in disguise,^12^ while in the Cotard syndrome the patients deny their own existence or that of a particular part of their body.^15^ Intermetamorphosis involves a belief that people around the patient switch places entirely, reverse-intermetamorphosis involves the belief that another person has replaced the patient both physically and mentally, and misidentification of reflection involves a belief that the reflection in the mirror is not of the patient but of a different person resembling the patient.^1^ Our patient showed features of both Capgras Syndrome and Reduplicative paramnesia when he stated that his wife had been substituted by a perfect copy of herself, the house he lived in only resembled his original property, and the city's most famous avenue had been duplicated in two identical copies.

Epidemiological studies of the prevalence of misidentification syndromes are scarce but show a prevalence of 0.12% in the general population. Capgras' syndrome has a high incidence (15%) in patients diagnosed with schizophrenia and also has an incidence of 2%-30% in those with Alzheimer's disease.^16^ The etiology of DMS and its subtypes varies in different populations studied but it is typically associated with psychiatric illness and neurological disorders, especially neurodegenerative diseases and particularly Lewy body dementia.^17^ Nonetheless, several other focal and diffuse neurological conditions have been associated with DMS in smaller studies or case reports including Parkinson disease, right hemispheric stroke,^15^ neurosyphilis,^18^ and hypothyroidism.^19^ Therefore, it is crucial to rule out a possible organic etiology when the onset is at an older age.^17^ To our knowledge, there are no reports of DMS directly associated with LE or lymphoma in the medical literature. We found only a few case reports linking DMS to other neurological autoimmune diseases. Sharma et al. reported the case of a 45-year-old woman with previously diagnosed Multiple Sclerosis (MS) that presented with an acute onset of psychotic symptoms compatible with Capgras Syndrome. Brain MRI of the patient showed single enhancing lesion in the right frontal lobe. Considered a relapse of MS, she was treated with immunoglobulin in association with Aripiprazole and showed significant clinical improvement.^5^ Sidoti et al. described the case of a 36-year-old female that, after treatment with corticosteroids for MS relapse, developed a paranoid disorder characterized by persecutory delusion (illusion of double) towards her husband. Brain MRI showed major active lesions compatible with MS but no injuries in areas associated with Capgras Syndrome were noted. The patient's neuropsychiatric condition improved with glatiramer acetate and quetiapine.^20^ Lebert et al. also reported early onset of Capgras Syndrome in a 40-year-old woman with MS, who demonstrated hypoperfusion in the right parietal cortex on single photon emission computed tomography.^21^ Hudson et al. presented the case of a 64-year-old man with Morvan's syndrome that acutely developed reduplicative paramnesia and markedly improved after a 5-day course of immune globulin and prednisone. Morvan's syndrome is a rare autoimmune disease usually associated with voltage gated potassium channel antibodies and characterized by peripheral nervous system hyperexcitability (myokymia and neuromyotonia), hyperhydrosis, sleep disorder, limb paresthesias, and encephalopathy.^22^

There is no consensus on specific lesion topography responsible for the clinical manifestations of DMS, but it is believed to arise from structural lesions of the very complex face processing areas,^23^ as well as from a disconnection between the temporal cortex and the limbic system.^15^ In a review article, Devinsky et al. claimed that misidentification syndromes, particularly reduplicative paramnesia and Capgras syndrome, result from neurologic damage to bifrontal lobes and/or right hemisphere of the brain. A dual mechanism is postulated for the delusional misidentification syndromes. Negative effects from right hemisphere and frontal lobe dysfunction result in impairment in self-monitoring, ego boundaries, attachment of emotional valence and familiarity to stimuli. Meanwhile, positive effects from release and overactivity of preserved left hemisphere areas lead to excessive and false explanations characterized by delusions seen in DMS.^24^

There are no formal guidelines currently outlining the standards for assessment or treatment of these syndromes. Once identified, organic, structural, and metabolic conditions must be ruled out and adequately treated. Comorbid substance use must also be identified. Routine blood work should address metabolic parameters of hepatic and renal function (to identify causes or indicators of delirium), thyroid function and when pertinent, levels of drugs such as lithium. Neuropsychological testing can be conducted, as deemed necessary.^3^ Brain imaging may help identify some neurological pathologies, such as stroke and multiple sclerosis. Neuroimaging studies most often reveal diffuse cerebral atrophy and/or bifrontal and right cerebral hemisphere.^25^ Diagnostic work-up also usually includes electroencephalogram findings.^16^ Several therapeutic approaches have been described and consist of antipsychotics, antidepressants for associated mood disorders, lithium for associated mania, group therapy and treatment of co-occurring psychiatric disorders, substance use, or medical disease. Case reports suggest that patients with DMSs who have an underlying organic etiology show remission on most occasions. The mainstay in symptomatic relief is the use of antipsychotics, and more recently atypical antipsychotics such as quetiapine have been increasingly prescribed to control Capgras' delusions.^16^ In a meta-analysis, Silva et al. examined 104 misidentification syndrome cases treated with antipsychotic medications, demonstrating that 70 patients showed improvement, whereas 34 did not.^26^ Prognosis of DMS is related to the etiology of the cause. If the cause is treatable, such as an infection or a depressive episode, the prognosis is more favorable. Patients with schizophrenia have a poorer prognosis. The prognosis in patients with dementia is less well understood. Medications can alleviate the symptoms, but relapses may occur with the progression of neurodegeneration.^16^

LE was initially identified as a paraneoplastic neurologic syndrome characterized by subacute onset of short-term memory loss, seizures, psychiatric changes, and neuroradiological or pathologic evidence of involvement of the amygdala and medial aspect of temporal lobes.^27^ Nowadays, LE is considered an autoimmune process that may or may not be associated with the presence of a tumor. The pathogenesis of the disease has been broadly categorized into two groups: associated with antibodies against intracellular neuronal antigens or with antibodies directed against cell membrane/extracellular antigens. Whereas the first group is frequently associated with cancers and responds poorly to treatment, the second group is less frequently associated with cancers and responds favorably to immunotherapy.^28^^,^ 29 Magnetic Resonance Imaging discloses high MRI T2 and FLAIR signal involving one or both medial temporal lobes while neuropathologic studies show dominant parenchymal infiltrates of T-cells supporting the hypothesis that classic paraneoplastic LE is mediated by a T-cell driven immune response.^30^ The diagnostic criteria of paraneoplastic LE formulated by Paraneoplastic Neurological Syndromes Euronetwork include: (i) subacute onset (days or up to 12 weeks) of seizures, short-term memory loss, confusion and psychiatric symptoms; (ii) neuropathological or neuroradiological evidence of involvement of the limbic system; (iii) exclusion of other possible etiologies of limbic dysfunction; and (iv) demonstration of a cancer within 5 years of the diagnosis of the neurological disorder or demonstration of a well characterized paraneoplastic antibody.^29^^,^ 31

Paraneoplastic LE is preferentially associated with small cell lung cancer (40%), germ cell tumors of the testis (20%), breast cancer (8%), Hodgkin's lymphoma, thymoma, and immature teratoma.^32^ The association between LE and Hodgkin's lymphoma is relatively well characterized and termed Ophelia Syndrome, while LE is extremely rare in patients with NHL, with only a few cases described.^33^ Mihara et al. reported a 59-year-old woman with paraneoplastic LE associated with diffuse large B-cell lymphoma but no immunotherapy or long-term follow-up were reported.^8^ Dögel et al. reported two additional patients, in whom malignant non-Hodgkin lymphomas of the B- and T-cell lines were detected. Treatment with corticosteroids in one and chemotherapy in the other case were associated with clinical improvement.^34^ Semnic et at. presented a case of a young adult with complete clinical course of non-Hodgkin's lymphoma followed by diagnosis of paraneoplastic limbic encephalitis one year after chemotherapy. No immunotherapy was reported and the patient died a few months later.^35^ Kanemitsu et al. reported the case of a 63 year-old man that presented with LE later attributed to diffuse large B-cell lymphoma, but his neurological condition deteriorated even after immunoglobulin and chemotherapy.^36^ Kawashima et al. described the case of a 62 year-old male with initial presentation of LE diagnosed with diffuse large B-cell lymphoma after investigation. Tumor resection and chemotherapy were performed but clinical symptoms failed to improve.^37^ Ishihara et al. reported the case of 42 year-old patient that developed a complex paraneoplastic syndrome comprising LE, cerebellar degeneration and olivary pseudohypertrophy associated with T-cell type malignant lymphoma following biopsy of the cervical lymph node. The patient's neurological condition improved slightly after chemotherapy, but subsequently deteriorated even though complete remission of lymphoma was documented. The authors suggested that lesions in the central nervous system in paraneoplastic neurological syndromes may follow a course independent of the original malignant disease.^38^ Market et al. reported the case of a 63-year-old woman who presented with LE and was later found to have bilateral renal lymphoma. The patient's clinical and neurological status improved dramatically during the first cycle of chemotherapy and remained in remission 16 months after diagnosis.^39^ Thuerl et al. reported the case of a 26-year-old man diagnosed with precursor T-cell acute lymphoblastic leukemia who developed paraneoplastic LE and did not undergo immunotherapy. The patient died one month later because of a relapse of lymphoblastic leukemia plus multiorgan failure where LE was confirmed post-mortem.^40^

In conclusion, we described the extremely rare association of DMS and Capgras Syndrome with LE secondary to diffuse large B-cell lymphoma. Capgras syndrome is a complex disorder defined as misidentification of persons by a patient generally in the course of neurodegenerative or psychiatric disorders. It is rarely linked to other conditions such as autoimmune neurologic disease. LE is an autoimmune entity characterized by inflammatory disturbance of medial temporal lobes usually associated with cancer. Non-Hodgkin lymphomas associated with LE have only been described in a few case reports. To our knowledge, there are no previous publications describing DMS in the context of LE and lymphoma diagnosis.
